# Neuroinflammatory disorders of the brain and inner ear: a systematic review of auditory function in patients with migraine, multiple sclerosis, and neurodegeneration to support the idea of an innovative ‘window of discovery’

**DOI:** 10.3389/fneur.2023.1204132

**Published:** 2023-08-10

**Authors:** Arianna Di Stadio, Pietro De Luca, Nehzat Koohi, Diego Kaski, Massimo Ralli, Anja Giesemann, Hans-Peter Hartung, Marta Altieri, Daniela Messineo, Athanasia Warnecke, Teresa Frohman, Elliot M. Frohman

**Affiliations:** ^1^GF Ingrassia Department, University of Catania, Catania, Italy; ^2^IRCCS Santa Lucia, Rome, Italy; ^3^Head and Neck Department, San Giovanni-Addolorata Hospital, Rome, Italy; ^4^The UCL Queen Square Institute of Neurology, London, United Kingdom; ^5^Department of Sense Organs, University Sapienza, Rome, Italy; ^6^Department of Interventional Neuroradiologie, Hannover Medical School, Hannover, Germany; ^7^Klinik für Neurologie UKD Heinrich Heine Universität Düsseldorf, Düsseldorf, Germany; ^8^Department of Neurology, University Sapienza, Rome, Italy; ^9^Department of Radiology and Pathology, University Sapienza, Rome, Italy; ^10^Department of Otolaryngology-Head and Neck Surgery, Hannover Medical School, Hannover, Germany; ^11^Distinguished Senior Fellows (Sabbatical), Laboratory of Neuroimmunology of Professor Lawrence Steinman, Stanford University School of Medicine, Palo Alto, CA, United States

**Keywords:** inner ear, neuroinflammation, CSF, Parkinson’s disease, multiple sclerosis, headache

## Abstract

**Background:**

Hearing can be impaired in many neurological conditions and can even represent a forme *fruste* of specific disorders. Auditory function can be measured by either subjective or objective tests. Objective tests are more useful in identifying which auditory pathway (superior or inferior) is most affected by disease. The inner ear’s perilymphatic fluid communicates with the cerebrospinal fluid (CSF) via the cochlear aqueduct representing a window from which pathological changes in the contents of the CSF due to brain inflammation could, therefore, spread to and cause inflammation in the inner ear, damaging inner hair cells and leading to hearing impairment identifiable on tests of auditory function.

**Methods:**

A systematic review of the literature was performed, searching for papers with case–control studies that analyzed the hearing and migraine function in patients with neuro-inflammatory, neurodegenerative disorders. With data extracted from these papers, the risk of patients with neurological distortion product otoacoustic emission (DPOAE) was then calculated.

**Results:**

Patients with neurological disorders (headache, Parkinson’s disease, and multiple sclerosis) had a higher risk of having peripheral auditory deficits when compared to healthy individuals.

**Conclusion:**

Existing data lend credence to the hypothesis that inflammatory mediators transmitted via fluid exchange across this communication window, thereby represents a key pathobiological mechanism capable of culminating in hearing disturbances associated with neuroimmunological and neuroinflammatory disorders of the nervous system.

## 1. Introduction

**Figure 1:**
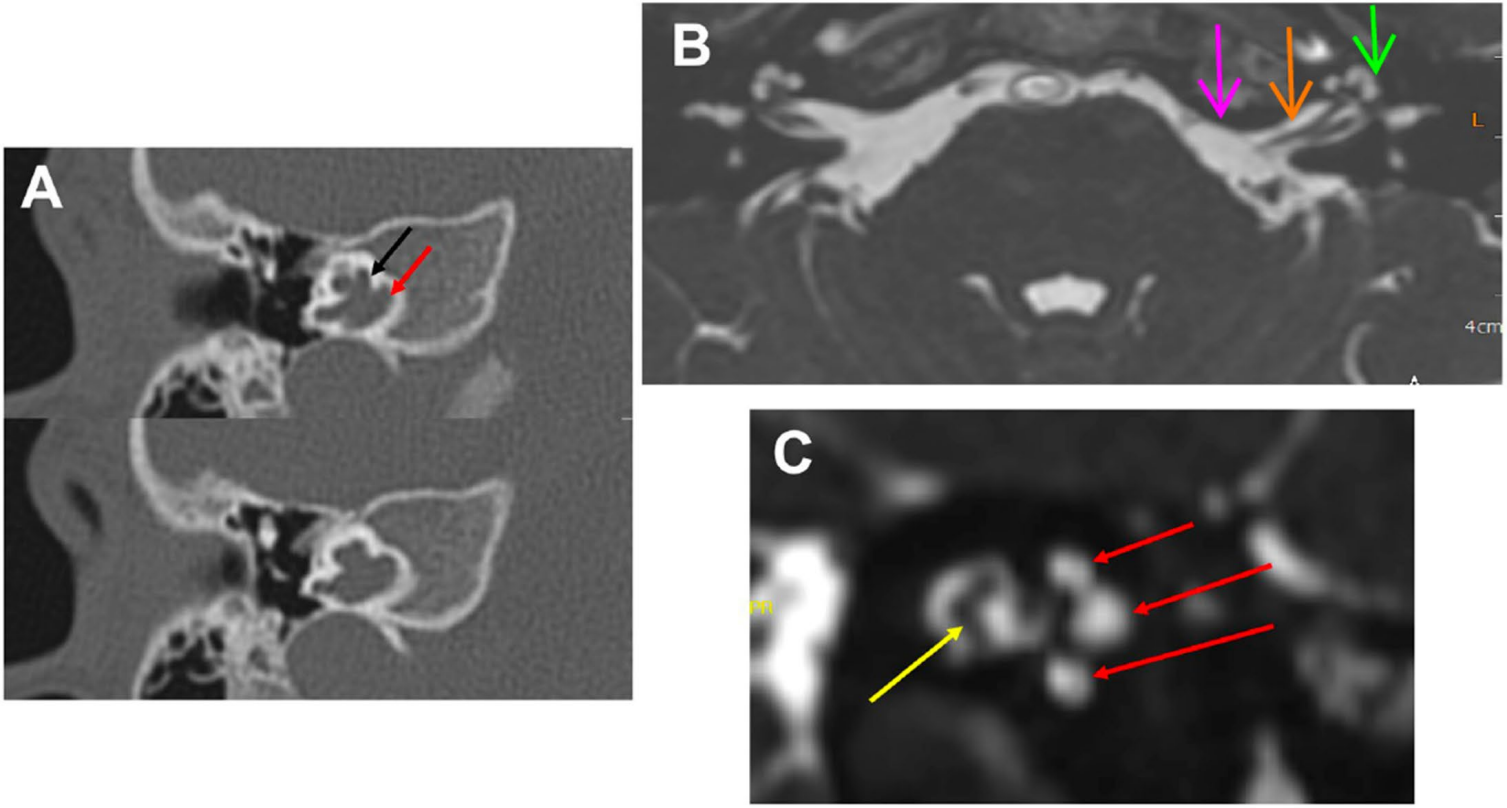
MRI and Inner Ear: **(A)** Computed tomography of the right temporal bone in a patient with of X-linked deafness. It shows an open connection between the CSF space and the perilymphatic space. The black arrow points to the basal turn of the cochlea and the red arrow points to the internal auditory canal. This is one possible malformation with a connection between these spaces. Others would be cases with a missing modiolus or defects in the modiolus itself. **(B)** MRI axial view of the brain, root entry zone of cranial nerve VIII (pink arrow), internal auditory canal (orange arrow), and inner ear (green arrow). The CSF (in white) goes through the internal auditory canal into the cochlea and vestibule (inner ear). **(C)** MRI parasagittal view of the internal auditory canal contains cochlear and vestibular nerves (yellow arrow) and CSF (in white); which infiltrates all three turns of the cochlea (red arrows).

The inner ear has three points of communication with the central nervous system (CNS): (i) the cochlear aqueduct; (ii) the internal auditory canals; and (iii) the vestibular aqueduct. The first two of these directly communicate with the subarachnoid space, and thereby the cerebrospinal fluid (CSF). The cochlear aqueduct allows communication between the CSF and the inner ear, given that the aqueduct opens into the scala tympani of the cochlear apparatus (Figure 1A). These communications can serve as a direct path for infections and inflammation to spread contiguously from the CSF to the inner ear and *vice-versa* (i.e. bidirectionally). This fluid exchange could explain why auditory symptoms are observed in several neurological diseases (1). While such auditory symptoms are usually attributed to a central pathology, inner ear involvement in neuroinflammatory conditions can also reflect peripheral pathophysiologic mechanisms. Auditory symptoms in neurological diseases are usually attributed to central pathology (3, 4).

Understanding whether the audiovestibular problem is of central or peripheral origin can be challenging. Standard audiological investigations such as pure tone audiometry (PTA) and speech perception threshold (SPT) provide limited information about the nature and origin of a patient’s hearing impairment (4, 5) especially if these tests are not accompanied by electrophysiological or imaging investigations (4). For instance, brain lesions can impair speech perception independent of the location of the injury and can even occur in the presence of a normal PTA, making the results of isolated testing insufficient to determine the presence or nature of audiological impairment, especially in patients with neuroinflammatory or neurodegenerative disorders.

Electrophysiological assessment allows objective measures of both central auditory processing (retro-cochlear) and cochlear hearing impairment (6). Auditory brainstem responses (ABR/BERA) are useful to assess the integrity of the auditory pathways from the cochlear nerve (its activity is reflected by wave I) to the brainstem nuclei in the midbrain (wave V shows the nuclei function) (6), therefore helping to identify lesions affecting the central auditory pathway in patients with neuroinflammatory [i.e., multiple sclerosis (MS)], cerebrovascular (i.e., stroke) and neurodegenerative [i.e., Alzheimer’s disease (AD)] disorders (6).

Otoacoustic emissions (OAE), either transient-evoked (TEOAE) or distortion product (DPOAE), measure the function of the inner ear through direct recording of outer (efferent) cochlear hair cells (OCH). Due to its high sensitivity, OAE can identify dysfunction of cochlear hair cells even in subclinical states (6). DPOAE is used to record auditory inner ear function in patients with neurological conditions (7–10). Accordingly, patients with MS (7), Parkinson’s disease (PD) (8), AD (9), and migraine (10) may show abnormalities in the test, reflecting OCH dysfunction, no unifying mechanism has yet been proposed to account for their abnormal DPOAE measures. Patients with these disorders may show cochlear OHC dysfunction (7–10).

To probe the hypothesis that the inner ear may be affected by neuroinflammation, we performed a systematic review to identify studies in which OAE (TEOAE and DPOAE) were utilized to explore inner ear integrity in patients with MS, PD, AD, and migraine. We postulate that neuroinflammation may play a role in the observed electrophysiological change, proposing the inner ear as a particular microcosm or window into cerebral pathophysiology.

## 2. Materials and methods

**Figure 2:**
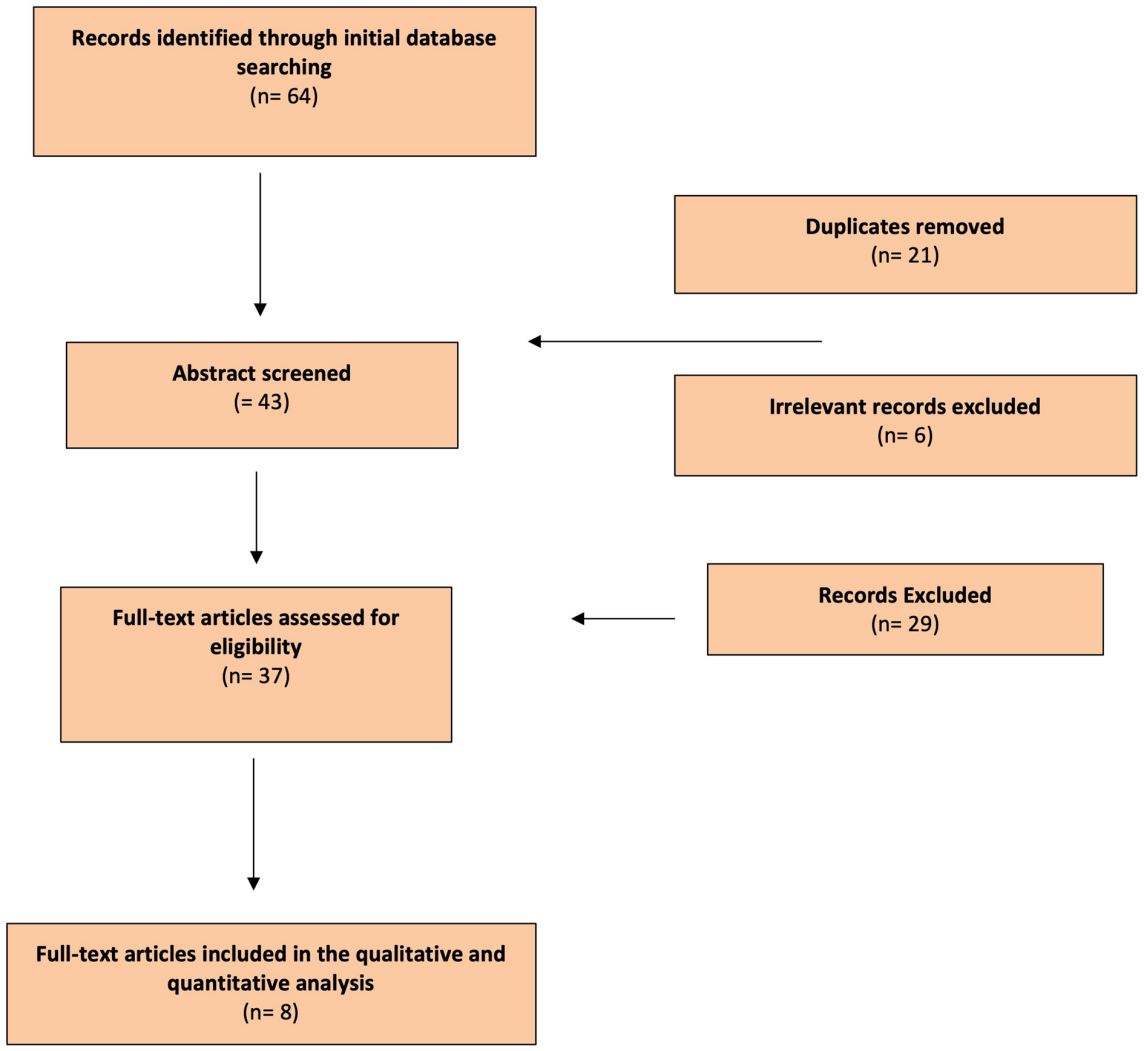
Flow Diagram: Literature search process (Preferred Reporting Items for Systematic Reviews and Meta-Analyzes flow diagram).

This study was performed in accordance with the Preferred Reporting Items for Systematic Reviews and Meta-analysis (PRISMA) checklist and statement recommendations (Figure 2). The nature of this review did not require Institutional Review Board approval.

### 2.1. Search strategy

A comprehensive search strategy, developed in partnership with a medical librarian, was performed on PubMed, Scopus, and Google Scholar without time restrictions. The keywords used were inner ear, OAE, DPOAE, TEOAE, neuroinflammation, inflammation, neurodegenerative disease, neuroinflammatory disease, Parkinson’s Disease (PD), Alzheimer’s Disease (AD), multiple sclerosis (MS), headache, migraine, microglia, and inflammatory cytokines. After initial screening, the keywords were combined to improve the specificity of the articles as follows: “OAE or DPOAE or TEOAE and neuroinflammation or neurodegeneration or MS or Parkinson Disease or AD or migraine.”

Four independent investigators (ADS, NK, PDL, and MR) reviewed the articles extracted from the literature review. Duplicates were removed and each reviewer singularly filled in an Excel data sheet (Microsoft Corporation, United States) including information extracted from the articles. Files were then compared and disagreements on the inclusion/exclusion papers were debated until complete agreement between researchers was achieved. Only papers that received full consensus were considered. PRISMA guidelines were followed to conduct the systematic review and the full list of references was screened for potentially relevant articles.

### 2.2. Study selection criteria

We included articles with the following characteristics: patients between 18 and 75 years of age who underwent OAE (DPOAE and/or TEOAE), and an established clinical diagnosis of migraine, PD, MS, or AD. Only articles in English with full text available were included. Selected articles were read in full to assess the study objectives and the level of evidence, and only relevant works were included in the study.

### 2.3. Data extraction

A spreadsheet was populated using the data extracted from the articles read in full by the researchers. The following information was included: name of the author, year of publication, type of study, country where the study was conducted, number of subjects analyzed, patients’ characteristics, results of PTA, tympanogram and impedance, presence/absence of OAE/DPOAE/TEOAE, treatment, outcome, presence or absence of the comparison group, and characteristics of control groups.

### 2.4. Statistical analysis

Using the patients included in the study and the healthy population, we calculated the odds ratio (OR) for alteration of OAE/TEOAE/DPOAE of neurological patients in general, and specifically of the patients suffering from MS, migraine, or PD.

### 2.5. Risk of bias assessment

The National Institutes of Health’s (NIH) quality assessment tools (case–control studies) were used because of the availability of risk-of-bias checklists for different study designs (11). The quality rating of each study was categorized as poor, fair, or good (i.e., unbiased, and fully described). Because of the particular nature of this study, we added another parameter of bias; those studies in which the audiological analyzes were incomplete (ie lack of tympanometry) were considered fair even if unbiased and fully described. Two authors independently determined the score for each article and disagreements were resolved by consensus among authors. The results are summarized in Table 1.

**Table 1:** Review results: characteristics of studies utilizing OAE measurements to explore inner ear integrity in patients with MS, PD, and migraine including rating.

References, year	Type of study	Sample size	Age (range), year; sex distribution	Disease	Audiological evaluation	Features of the control group (sample size; age median and range, year; and sex distribution)	Results of the study	Overall quality rating consensus
Saberi et al., (2012) (12)	Prospective cross-sectional	60	20–38 yr. (mean age: 29.86 ± 9.8 years); 44 women and 16 men	Multiple sclerosis	PTA, OAE (TEOAE and DPOAE), ABR Tympanometry not reported	38; 27–49 yr. (mean age: 31.39 ± 8.3 years); 27 women and 11 men	12.5% of case ears and 3.9% of the control ears had abnormal PTA (*p* = 0.043). In all, 62 ears (51.7%) in the case group and 44 ears (59.5%) in the control group had abnormal TEOAE (*p* = 0.29). Almost 34% of subjects in the case group and 66% in the control group had abnormal DPOAE (*p* = 0.000, the preference was for the control group). A total of 20% of case ears and 9.2% of the control ears had abnormal ABRs (*p* = 0.044), whereas 6.7% of case ears and 2.6% of control ears had retrocochlear abnormality (*p* = 0.181)	Fair
Mokbel et al., (2014) (13)	Prospective cross-sectional	34	21–59 yr. (Mean age: 38 ± 10.2); 23 women and 11 men	Vestibular migraine	PTA, Tympanometry, and OAE (only DPOAE)	30; 20–60 yr. (Mean age: 39.4 ± 9.9 years); 18 women and 12 men	The mean emission amplitudes across the DPOAE-measured frequencies in both ears of patients with definite vestibular migraine were lower than that of the normal subjects but statistically non-significant (*p* > 0.05)	Fair
Cameron and Sun, (2015) (14)	Prospective cross-sectional	7	22–26 yr.; 5 women and 2 men	Chronic migraine	Tympanometry, PTA, OAE (only DPOAE)	3; 22–26 yr	There was no significant difference in regular audiometry threshold between the migraine group and the control group. The amplitude of DPOAEs was reduced by more than 10 dB in 80% of subjects with chronic migraine in comparison to 30% of subjects in the control group (*p* < 0.05)	Poor
Shen et al., (2015) (15)	Prospective cross-sectional	23	18–52 yr. (Mean age: 34 ± 9 years); 7 women and 16 men	Tension-type headache (TTH)	PTA, OAE (TEOAE and DPOAE), ipsilateral and contralateral ART Tympanometry not reported	26; 24–51 yr. (Mean age: 35 ± 8 years); 9 women and 17 men	The TTH group showed higher thresholds (*P* < 0.05) for both pure tone and extended high-frequency audiometry at all frequencies except for 9, 14, and 16 kHz. All ART thresholds were significantly higher (*p* < 0.05) in the TTH group compared with the controls, except for the ipsilateral reflex at 1 kHz, but the threshold differences between the ASR and the corresponding pure tone audiometry did not differ (*p* > 0.05) For the DPOAEs, the detected rates were lower (*p* < 0.05) in the TTH group compared with the controls at 4 and 6 kHz, and the amplitudes and signal-to-noise ratio (S/N) were not significantly different between groups No differences in the TEOAEs (*p* > 0.05) were observed for the detected rates, amplitudes, S/Ns, or contralateral suppression, except for the S/Ns of the 0.5–1 kHz TEOAE responses, which were significantly higher (*p* < 0.05) in the TTH group	**Fair**
Pisani et al., (2015) (8)	Prospective cross-sectional	11	59–71 yr. (Mean age) 66.5 ± 7.5 years; 8 men and 3 women	Parkinson’s disease	Tympanometry, PTA, OAE (TEOAE and DPOAE)	8; 55–74 yr. (Mean: 66.0 ± 8.8 years); 4 women and 4 men	At pure-tone audiometry, higher auditory threshold levels were observed in PD when compared to the controls. DPOAEs demonstrated an overall impairment of cochlea across all frequencies, suggesting a subclinical dysfunction in PD patients.	Good
Xue et al., (2020) (18)	Prospective cross-sectional	46 (Group 1: 28 patients with migraine. Group 2:18 patients with vestibular migraine)	Group 1: 16–62 yr. (Mean age: 34.66 ± 9.61 years); 20 women and 8 men Group 2: 19–57 yr. (Mean age: 36.25 ± 10.70 years); 15 women and 3 men	Migraine and vestibular migraine (VM)	PTA, OAE (only DPOAE), ABR Tympanometry not reported	25; 25–52 yr. (Mean age: 32.64 ± 7.71 years); No available info	The pure tone in the VM group showed higher thresholds at lower frequencies (250, 500, 1,000, 2,000 Hz) than the control group, with statistical differences observed (P250 Hz = 0.001, P500 Hz = 0.003, P1,000 Hz = 0.016, P2,000 Hz = 0.002) Compared with the healthy controls, the patients with VM had significantly lower amplitudes of DPOAE at 1 kHz (*p* < 0.001) and 2 kHz (*p* = 0.020), and the patients with migraine had lower amplitudes at 2 kHz (*p* = 0.042). Compared with the control group, the patients with migraine reported prolonged latency of wave V (*p* = 0.016) and IPL I–V (*p* = 0.003)	**Fair**
Di Mauro et al., (2019) (16)	Prospective	45 patients newly diagnosed with relapsing–remitting MS	Mean age: 37 ± 8 years; 18 men and 27 women	Multiple sclerosis	PTA, OAE (TEOAEs, DPOAE) and ABR Tympanometry not reported	48 healthy matched for sex and age. Mean age: 35 ± 8 years; 19 men and 29 women	Auditory brainstem response and pure tone audiometry thresholds resulted within the normal range in all patients. The amplitudes of transient-evoked and distortion-product otoacoustic emissions responses were significantly reduced at 1000, 1500, 2000, and 3,000 Hz in the study group compared to the control group	**Fair**
Sisto et al., (2020) (19)	Prospective cross-sectional	66	56–74 yr. (Mean age: 64 ± 9 years); 33 women and 33 men	Parkinson’s disease	PTA, DPOAE Tympanometry not reported	31; 54–74 yr. (Mean age: 62 ± 10 years); 16 women and 15 men	Significant asymmetry was found in the auditory function, as both otoacoustic responses and audiometric hearing levels were worse in the ipsilateral ear Significantly worse hearing function was also observed in patients with PD compared to controls, confirming previous studies	**Fair**

PTA, pre-tone audiometry; OAE, otoacoustic emissions; DPOAE, distortion product otoacoustic emissions; TEOAE, transient-evoked otoacoustic emissions; PD, Parkinson’s disease; VM, vestibular migraine; ABR, auditory brainstem responses; TTH, Tension-type headache; ART, acoustic reflex thresholds; MS, Multiple sclerosis; IPL, interpeak latencies. Bold: studies that were considered fair due to a lack of information about tympanometry results.

## 3. Results

Eight articles met the study selection criteria and were included in this systematic review (Table 1) (8, 12–19). The risk of bias is shown in Table 1.

Of these, four articles analyzed OAE in patients with headache disorders (13–15, 18), two studies evaluated patients with PD (8, 9), and only two considered findings in MS. (7, 16) We did not find any articles that analyzed OAE in patients affected by AD. The study exploring auditory function in patients with ‘migraine’ included a variety of migraine and headache subtypes: one study analyzed patients with vestibular migraine (13), one with cephalgic migraine (14), one study included both acephalgic and vestibular migraine patients (18), and one study analyzed auditory function in patients with tension-type headache (15). PTA associated with OAE was performed in all studies; three studies also reported the results of tympanometry. Note: regarding this last finding, because tympanometry is routinely performed in a clinical setting, especially before DPOAE, it is possible that when tympanometry results were not present in the study it was due to an omission from the authors. For this reason, the correct term is ‘not reported’ rather than ‘not performed’.

All included studies used DPOAEs, four of these TEOAEs and DPOAEs; only three studies (12, 17, 18) included ABR data.

Eight studies included a total of 291 patients and 209 healthy controls. Of these, seven identified changes in the OAEs in patients affected by a neurological disease (MS, migraine subtypes, and PD) when compared to healthy controls. Only a single study (*n* = 60), which was the oldest study (2012) analyzed, did not show any differences in audiological function between patients with MS and healthy control participants.

Calculation of odds ratio (OR) showed that, in general, patients with neurological diseases had a significantly increased risk of presenting with alterations in the DPOAE (OR:14.3673; CI 95%: 8.1 to 25.3; *p* < 0.0001) when compared with healthy individuals, based on a total of 291 neurological patients and 209 healthy controls. At the individual disease level, patients with migraine/headache (109 patients) had a significantly increased risk of presenting with altered DPOAE (OR:12191; CI 95%: 490 to 303,083; *p* < 0.0001) when compared to healthy controls. Patients with PD (*n* = 77) had a significantly increased risk of showing altered DPOAE (OR:12245; CI 95%: 238 to 628,792; *p* < 0.0001) when compared with age-matched healthy controls. Finally, patients with MS (105 patients) also presented an increased risk of having an alteration in the results of DPOAE (OR:3.96; CI 95%: 2.1 to 7.3; *p* < 0.0001) when compared with a healthy population.

## 4. Discussion

**Figure 3:**
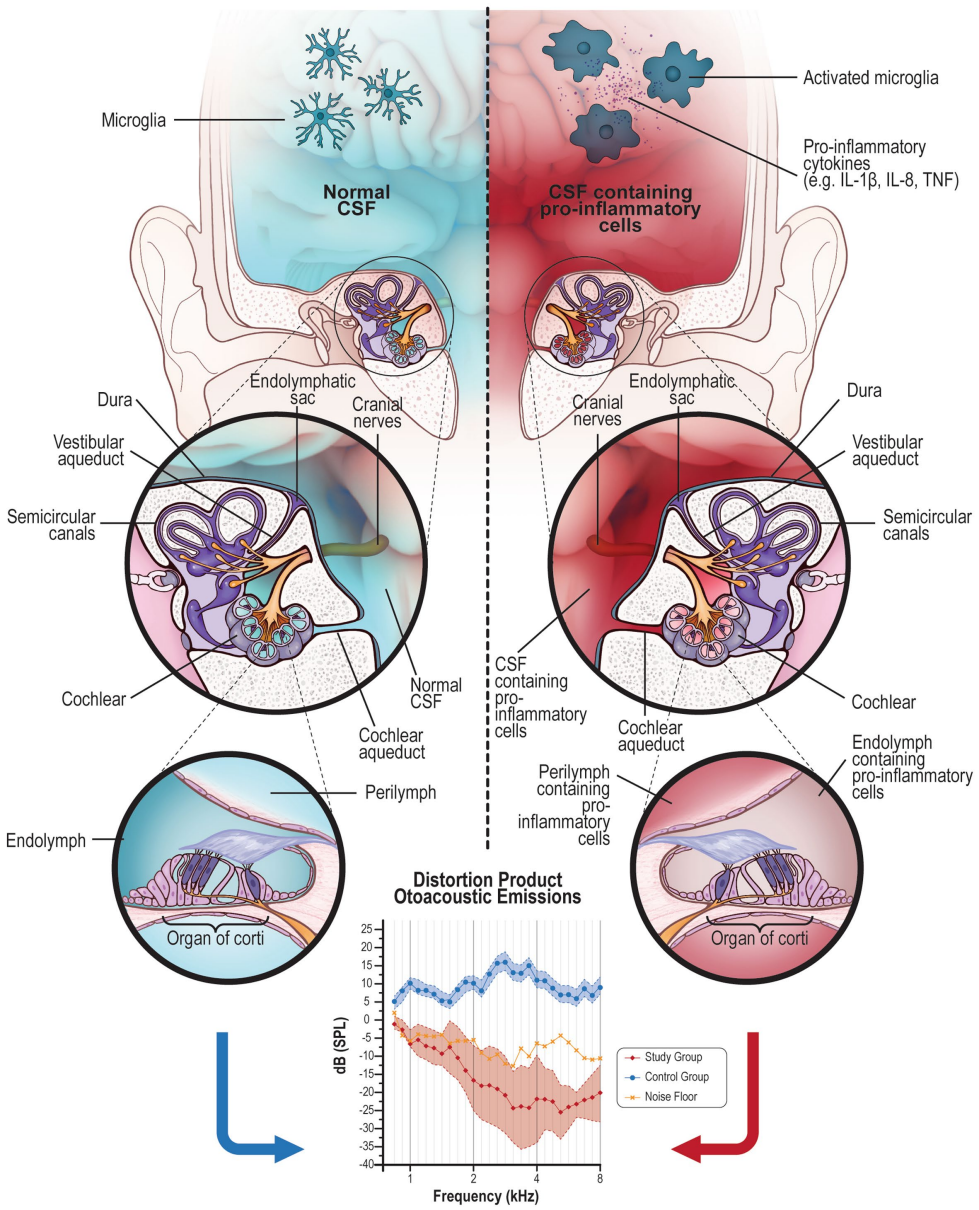
The liquid exchange: Right side: CSF (in blue) contained in the brain can normally pass into the inner ear *via* the cochlear aqueduct or through the internal auditory canal reaching the cochlea, sharing its content with the perilymph. On the other side, even the perilymph can retrogradely share some elements with CSF, although the exchange is limited by the small content of the liquid in the inner ear. This is a physiologic mechanism of fluid mobilization between these two organs. Normal CSF contains microglia in a resting state. Left side: In case of brain inflammation, microglia are activated in their pro-inflammatory (M1) phenotype that produces pro-inflammatory elements. These cells and their product contained in the CSF (in red) could migrate into the inner ear (perilymph) causing inflammation of the organ of Corti. Generally, a high concentration of pro-inflammatory elements is necessary to cause a central symptom; on the contrary, even a few elements in a small area, such as the inner ear, can be deleterious for the cells. This inflammation alters the normal function of the inner and outer hair cells; DPOAE altered or absent response indicates damage to the outer hair cells.

We observed that patients with neurological diseases had an increased risk of presenting altered results in OAE without suffering from manifest hearing impairment when compared with healthy individuals. Despite the different clinical presentations and presumed underlying neuronal mechanisms underpinning these neurological disorders (MS, PD, and migraine), there is emerging evidence to suggest neuroinflammation may be a common hallmark. For example, pro-inflammatory elements have been found in the CSF of patients with MS (20) and the role of these elements is under investigation in PD (21), allowing us to speculate that alteration in the OAE may be a harbinger of MS and PD (Figure 3).

A higher OR for altered OAE was observed in patients with migraine/headache when compared to patients with PD and MS; perhaps because of the higher concentration of pro-inflammatory cytokines in the CSF of patients with migraine rather than in the CSF of patients with PD (22) or MS. (20) In PD, neuroinflammation tends to be chronic and the level of pro-inflammatory cytokines stabilizes over time (23); on the other hand, many patients with MS are treated with disease-modifying therapeutics that considerably reduce active neuroinflammation (24, 25).

### 4.1. Migraine and auditory findings

The use of OAE to assess auditory function has been applied in patients suffering from migraine subtypes. Patients with tension-type headaches showed subclinical changes in cochlear function when examined with PTA and OAE (TEOAE and DPOAE), according to the results from Shen et al. (15). Cochlear function was also found to be affected in patients with chronic migraine, in which the amplitude of DPOAE was reduced by more than 10 dB (14). Investigation of the peripheral and central auditory systems in patients with vestibular migraine showed that both systems were involved (18). These results agree with the findings of other researchers (13), who stated that patients with vestibular migraine and normal peripheral hearing sensitivity may have subclinical cochlear derangements associated with the disease. A recent study identified clear signs of neuroinflammation in patients affected by migraine using PET and MRI imaging (26). It has been proposed that neuroinflammation, if not adequately treated, might lead to neurodegeneration; the authors proposed some molecules that improve mitochondria metabolism ameliorating ATP production and ROS removal. In the authors’ opinion, the improvement of mitochondrial function could be helpful to indirectly stimulate the polarization of microglia in a “good” (M2) state and promote neuro-regeneration (27). However, these intriguing results have, to our knowledge, not yet been replicated by others.

### 4.2. Multiple sclerosis and auditory functions

The prevalence of hearing loss in a cohort of patients with MS has been evaluated and compared with the audiological results (PTA, OAE, and ABR) of a healthy control group (12). Patients with MS were more likely to show abnormal PTA (12.5% vs. 3.9%; *p* = 0.043). High frequency-PTA and two modalities of OAE were not significantly different between case and control ears. The absolute latencies of waves I, II & V did not show any significant differences, but 10 and 11.7% of case ears had increased interpeak latencies of I-III, whereas 1.3% and none of the control ears had increased interpeak latencies of III-V, concluding that hearing loss is more common in patients with MS ears, especially when determined by using PTA and ABR.

Subclinical alterations of auditory function were identified using both TEOAE and DPOAE in a sample of patients newly diagnosed with relapsing remitting MS. (16) The researchers also performed ABR to study the retrocochlear auditory pathway. They identified normal ABR and PTA but a reduced amplitude of TEOAE/DPOAE at 1000, 1500, 2000, and 3,000 Hz in patients with MS compared to the control group.

Di Stadio and Ralli (3) proposed that the inner ear could be affected by the same neuroinflammatory phenomena that affect the brain in patients with MS; the authors were promoters of the concept that the inner ear could be the first locus to manifest the signs of the disease because microglia and/or macrophages can migrate in this organ and destroy hair cells (28–30). Microglia, which exert pro-inflammatory mechanisms when activated (e.g., the M1 microglial phenotype) in MS, could lead to immune-mediated destruction and clearance of hair cells (phagocytosis), and indirectly through the production of reactive oxygen species (ROS) (neuroinflammation) (3, 31).

A recent study showed that patients with MS (3) may manifest auditory symptoms more frequently than was previously reported in the literature (32); the latter study identified a very low prevalence of auditory disorders in patients with MS. However, this retrospective study was associated with a bias given that the investigators principally focused on patients who were admitted to the emergency room for sudden hearing loss, and not on the prevalence of auditory impairment specifically in the MS population. Because sudden hearing loss can affect all people, and for a diverse heterogeneity of underlying causes, the absence of stratification for the disease could induce an underestimation of auditory disorders in the specific population of MS. (2–33)

The passage of pro-inflammatory cytokines or cellular elements from the CSF into the inner ear (3) can selectively destroy the hair cells, a mechanism that may account for the results reported by Di Mauro et al. (16). The authors found alterations of DPOAE in the absence of manifest hearing loss in patients with MS. They hypothesized that alterations of DPOAE might be a subclinical signature of active neuroinflammation in MS subjects.

### 4.3. Parkinson’s disease and auditory findings

In 2015, a team of Italian researchers demonstrated that otoacoustic emission recording and PTA identified levodopa-sensitive cochlear dysfunction in patients with PD. (8) They proposed the use of objective DPOAE responses for monitoring therapeutic responses during the disease. These results have been confirmed in another study performed in 2018 (17). Furthermore, a recent publication (2020) quantified hearing function in a group of patients with PD and found a significant asymmetry in auditory function correlated with PD lateralization, which appeared to be worse in the ipsilateral ear, suggesting the possible diagnostic use of cochlear dysfunction asymmetry determined by otoacoustic responses as a biomarker of PD. (19)

Whether a similar mechanism to the one proposed for MS may account for DPOAE changes in patients with PD remains to be elucidated.

### 4.4. The liquid exchange can explain the auditory alteration

The relative cellular constituents of perilymph and CSF has been widely studied (34–36) to understand their relationship and common characteristics. The inner ear has a barrier similar to the brain blood barrier (BBB) called blood perilymph/labyrinth barrier (BPB), which is different from the BBB only for the lack of astrocytes (34); these barriers facilitate liquid exchange and protect the brain and inner ear from the entry of blood-borne infection (septicemia) (34). The potential communication channels between CSF and perilymph can be the cochlear aqueduct, the pores in the cochlear modiolus, and the perineural space in each singular modiolus canal (Figure 3).

The cochlear aqueduct is totally occluded in 7% of the adult population, filled with very soft tissue in 59%, and open in 34% of the adult population (37), so this communication is not always present. Alternatively, the porous structure on the surface of the modiolus may allow communication between the perilymph and the perivascular and perineural spaces (35). Each singular canal contains a nerve branch and through the perineural space of this canal, the perilymph could communicate with the posterior part of the internal auditory canal (IAC) through the perineural space (35). A connection between these two liquids has been ultimately confirmed by the passage of drugs from the inner ear into the brain (34–36). It follows that if a drug injected in the perilymph passes from the inner ear into the brain, it is equally possible that pro-inflammatory elements pass from the brain into the inner ear through the CSF.

The prolonged presence of pro-inflammatory elements within the inner ear might initially cause transitory symptoms, i.e., tinnitus (33), later progressing to more definitive hearing loss identifiable on PTA and suggesting hair cell damage (1). Indeed, Warnecke et al. (38) found the presence of pro-inflammatory cytokines in the perilymph of patients affected by severe hearing loss (HL). Although the pro-inflammatory cytokines identified by the authors can be produced by the hair cells themselves because they commonly release these elements in cases of noise exposure (39). The researchers suggested pro-inflammatory cytokines and chemokines recruit pro-inflammatory leukocytes (and macrophages), which can then foment damage to inner ear architecture. The authors concluded that inflammation itself can explain the death of hair cells.

Pro-inflammatory cytokines and inflammatory elements contained in the CSF might also, because of liquid exchange, damage both stria vascularis (SV) and the spiral ligament (SV) (also called the cochlear lateral wall) (40). The alterations of the functions of these two structures indirectly impact the results of the OEA and might explain the alterations observed in patients with neuro-inflammatory disorders. These structures, as well as the hair cells, might be damaged by the pro-inflammatory elements that migrate from the CSF into the inner ear through the perilymph; the damaged SV loses its capacity of removing metabolites into the organ of Corti, ulteriorly worsening the inner ear environment and causing the sufferance/death of the outer hair cells, which is detectable by OAE (41).

There is evidence to suggest that neuroinflammation is common in migraine (26), PD (42), and MS (43); pro-inflammatory cytokines can be found in the CSF in all these conditions (44) and, although the neuro-inflammation is of different origin and cause (45–47), these diseases share common typical findings such as the presence of microglia (45–47).

Because CSF and perilymph within the inner ear are connected by the cochlear aqueduct in some patients, while in others the connection is directly by liquid exchange through the internal auditory canal (IAC) (Figure 1), pro-inflammatory elements could pass between these two fluid reservoirs leading to damage to hair cells. Monitoring the changes in DPOAE could be useful to detect an early relapsing event in MS (1, 16) and to indirectly evaluate the efficacy of anti-neuroinflammatory treatments (48). Consequently, an improvement in outer hair cell function might indicate reduced inflammation, as an indirect measure of neuroinflammation in the brain.

While DPOAE only tests the function of the outer hair cells (OHC), there is recent evidence that damage to the inner hair cells (IHC) may precede damage and functional disruption of the OHC (49, 50); the potential implication being that ‘hidden hearing loss’ may be derived from discrete damage to the IHC.

Animal models of CNS inflammatory demyelination, (i.e., using experimental autoimmune encephalomyelitis (EAE)) have identified pro-inflammatory cells in the inner ear. Examining human temporal bones to identify the presence of microglia (20) and collecting perilymph during cochlear implantation in patients with concomitant PD or MS, will be necessary to confirm the hypothesis of the inner ear as a microcosm of the brain (macrocosm). Such an approach is similar to what has already been demonstrated for the retina and the optic nerve, in particular, as a window for investigating the CNS in general (51). Moreover, investigating the integrity of the auditory system by OAE in patients affected by MS, PD, and AD may become of pragmatic and diagnostic relevance, given that even subtle and highly discrete alterations of inner ear hair cells might constitute a harbinger of active neuroinflammation (52).

#### 4.4.1. Study limitations

This study presents several limitations. First, there is an imbalance between the number of studies identified and analyzed that could affect the OR. Second, the studies included in the analysis utilized different auditory recording devices, which may affect the reproducibility across studies. Due to the small number of studies that assessed DPOAE/TEOAE in neurodegenerative/neuroinflammatory disorders, we were obliged to include in this systematic review studies that share “neuro-inflammation” as a possible neuropathological process (MS, PD, and migraine), accepting that these may have important pathological heterogeneity. Along these lines, these diseases have different forms of neuro-inflammation and different balances between neuroinflammatory and neurodegenerative phenomena. Studies focused specifically on a single disease, i.e., MS or PD, are necessary to confirm this hypothesis. Importantly, none of the studies included in this review reported specific data relating to the neuro-immunological profile of the patients, including the degree or severity of the neuroinflammation. This weakness underlines the need for robust studies exploring auditory function in patients with MS, PD, and AD. Another major limitation is that only three studies reported tympanometry results and because an altered tympanometry can affect the results of OAE/TEOAE/DPOAE the lack of this information needs to be taken into account. Moreover, all studies had small sample sizes and given the potential for variability in the auditory function outcomes, the results should be confirmed on larger patient cohorts. Finally, the relationship between neuroinflammatory mechanisms to the corresponding electrophysiological signatures of cochlear hair cell dysfunction in those with specific neurological disorders remains speculative.

## 5. The importance of early diagnosis

An early diagnosis using non-invasive tests helps to reduce the risk of spreading neuroinflammation and provides an opportunity to treat patients earlier, for example in diseases with a relapsing phase such as MS. (43, 45) We also know that new drugs for stopping neuroinflammation are currently under-study (46–48) and a non-invasive test such as DPOAE could be a screening tool both in normal and at-risk populations (people with a familial history of neuroinflammatory/neurodegenerative diseases), allowing earlier treatment and potentially avoiding the deterioration of neuroinflammation or neurodegeneration (53, 54).

Like the development of objective methods to assess both structure and function in the visual system (51), such as optical coherence tomography (OCT), multifocal visual evoked potentials, multifocal electroretinography, and infrared pupillometer, methods to assay inner ear function and inner ear methods are now similarly emerging that may prove to be excellent biomarkers to diagnose, treat, and follow the course of patients with inflammatory disease.

## 6. Conclusion

The results of this systematic review suggest that patients with migraine, MS, and PD have an increased risk of presenting altered OAE compared to controls. From this perspective and considering some shared neuro-inflammatory profiles, we speculate that pro-inflammatory cytokines could pass from the CSF to the inner ear, with OHC damage representing an early step in the sequential cascade of inner ear inflammatory disease. TEOAE and perhaps even DPOAE responses and their correlation with CSF findings may help to elucidate active mechanisms of neuroinflammation during the subclinical phases of CNS neurodegenerative disorders. We hypothesize that the inner ear might be a novel anatomical entity from which to more fully characterize the pathophysiology of brain diseases, but also a “window” of opportunity for early detection and treatment when the burden of such disorders is small and potentially reversible.

## Data availability statement

The raw data supporting the conclusions of this article will be made available by the authors, without undue reservation.

## Author contributions

AS: conceptualization, writing the paper, analyzes and review of data, definition of conclusions, supervision, and original idea. AS, PL, and MR: data search. AS, DM, MA, and AG: statistical analysis. AS, PL, NK, DK, AW, TF, and EF: manuscript writing. AS, PL, MR, and H-PH: figures and tables editing. AS, TF, EF, AW, and H-PH: manuscript revision. AS, TF, and EF: supervision. All authors contributed to the article and approved the submitted version.

## Funding

This study was supported by current research funds of Santa Lucia Research Hospital of Rome.

## Conflict of interest

The authors declare that the research was conducted in the absence of any commercial or financial relationships that could be construed as a potential conflict of interest.

## Publisher’s note

All claims expressed in this article are solely those of the authors and do not necessarily represent those of their affiliated organizations, or those of the publisher, the editors and the reviewers. Any product that may be evaluated in this article, or claim that may be made by its manufacturer, is not guaranteed or endorsed by the publisher.
